# Silicosis initially presenting with empyema

**DOI:** 10.1093/occmed/kqae047

**Published:** 2024-06-10

**Authors:** C Reynolds, C Ross, P Cullinan, P Blanc

**Affiliations:** National Heart and Lung Institute, Imperial College London, London, UK; Imperial College Healthcare NHS Trust, London, UK; Imperial College Healthcare NHS Trust, London, UK; National Heart and Lung Institute, Imperial College London, London, UK; University of California San Francisco School of Medicine, San Francisco, California, USA

## Abstract

The current global outbreak of artificial stone silicosis is a recrudescence of a major occupational disease in the context of a novel exposure source. Respirable crystalline silica exposure, even without frank pneumoconiosis, is associated with an increased risk of respiratory infection. Empyema is a well-recognized complication of bacterial pneumonia; pneumonia among working-age adults, in turn, has been epidemiologically linked to occupational exposure to fumes and dust, including silica. A connection between empyema and silica dust inhalation has not been reported, however, whether through antecedent pneumonia or another mechanism. We describe a case of silicosis initially presenting with empyema in a 31-year-old Computerized Numerical Control stone-cutting machine operator who had heavy exposure to artificial stone and other rock dust.

## Teaser

Occupational exposure to respirable crystalline silica is well known to increase the risks of pulmonary tuberculosis and non-tuberculous mycobacterium infections. Its relationship to the risk of pneumococcal pneumonia and sequelae such as empyema is less well known. We describe a case of silicosis initially presenting with empyema in an artificial stone worker and summarize the related literature.

**Key learning points**

**What is already known about this subject:**

- There is an ongoing worldwide outbreak of silicosis associated with the use of high-silica-content artificial stone countertops and other products.
- Respirable crystalline silica exposure, even without frank pneumoconiosis, is associated with an increased risk of respiratory infection.

**What this study adds:**

- Awareness of the potential for respirable crystalline silica exposure to increase risk of respiratory infection, and sequelae of respiratory infection including empyema, can help to identify silicosis cases.

**What impact this may have on practice, policy or procedure:**

- Further work is needed to understand the burden of infectious disease in workers exposed to respirable crystalline silica, as well as the relationship with silicosis, to inform prevention efforts.

## Background

The current global outbreak of artificial stone silicosis [1] is a recrudescence of a major occupational disease in the context of a novel exposure source. Respirable crystalline silica exposure, even without frank pneumoconiosis, is associated with an increased risk of respiratory infection [2,3] as well as autoimmune disease. Empyema is a well-recognized complication of bacterial pneumonia; pneumonia among working-age adults, in turn, has been epidemiologically linked to occupational exposure to fumes and dust, including silica [4]. A connection between empyema and silica dust inhalation has not been reported, however, whether through antecedent pneumonia or another mechanism. We describe a case of silicosis initially presenting with empyema, linked to exposure to artificial stone and other rock dusts.

## Case presentation

A 31-year-old previously healthy male presented to the emergency department complaining of productive cough, subjective fever and right-sided chest wall pain. His temperature was 36.8°C oral; pulse, 108 beats per minute; oxygen saturation at rest, 97% on room air; and blood pressure, 138/79 mmHg. Physical examination did not reveal adventitious breath sounds or dullness to percussion.

## Investigations

**Figure 1:**
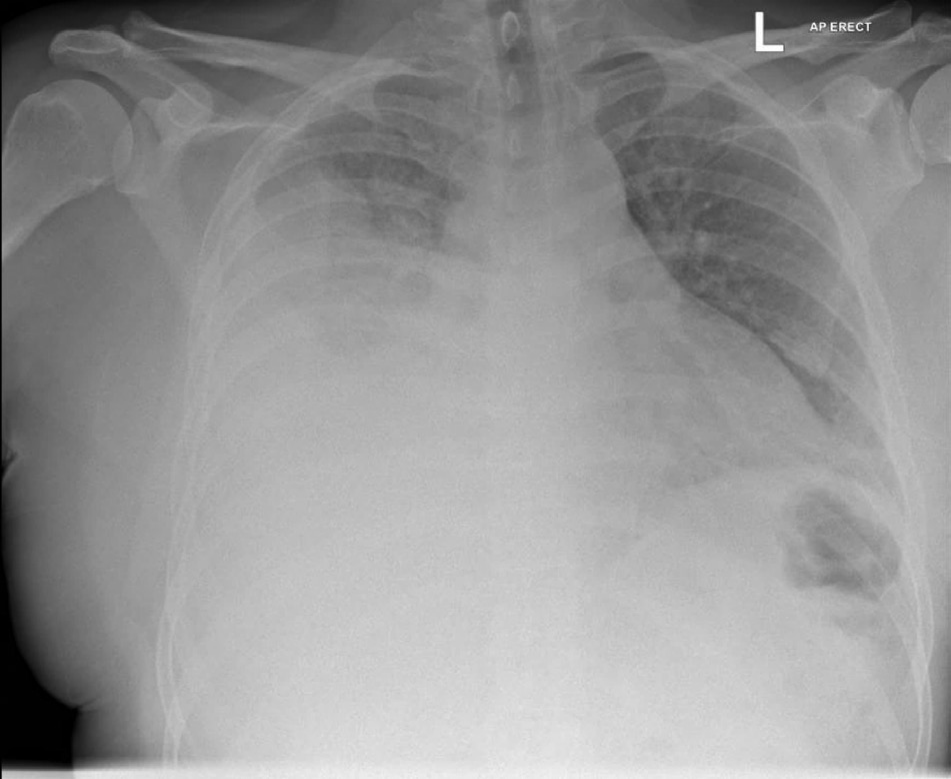
Chest radiograph on day of admission to hospital (day 4) showing right-sided opacification

His white cell count was 15 × 10^9^/L (neutrophils 11 × 10^9^/L) and his C-reactive protein (CRP) was 146 mg/L. A posterior–anterior view chest radiograph was interpreted as showing right basal consolidation and a small right-sided effusion. He was diagnosed with pneumonia and managed with outpatient ceftriaxone intravenous infusions and oral clarithromycin. On day 2, his oral temperature was 38.3°C and day 3, 37.8°C. By day 4 of this regimen, the patient complained of worsening right-sided chest wall pain, his temperature was 37.8°C, pulse was 114 beats per minute, oxygen saturation was 92% at rest on room air and blood pressure 144/83 mmHg. By this time, his white cell count had climbed to 25 × 10^9^/L (neutrophil count 19 × 10^9^/L) and his CRP to 454 mg/L. Plain radiographic imaging showed right-sided opacification (Figure 1); CT imaging showed a right-sided loculated fluid collection in the pleural space with a thick rind and contrast enhancement, consistent with an empyema. There was extensive consolidation and collapse within the right lung, which was worst in the right lower lobe, and a 16-mm right paratracheal lymph node. The patient met the criteria for clinical sepsis and required intensive care for vasopressor support. Thoracic ultrasound in the intensive care unit showed findings consistent with dense consolidation and a complex, septated pleural effusion. Thoracocentesis with ongoing chest tube drainage yielded turbid yellow fluid (protein level 55 g/L, LDH 1632 units/L, glucose < 0.05 mmol/L); the fluid was too turbid for a cell differential to be performed. Cytology was consistent with acute inflammation, showing increased numbers of neutrophils, and some macrophages and lymphocytes. No organisms were seen on gram stain. Cytology was negative for malignant cells. Pleural, blood and sputum cultures, including acid-fast bacilli culture, were all negative. Pleural fluid 16sRNA and a standard bacterial DNA panel (which included streptococcus pneumoniae) were negative, as were urinary assays for legionella and pneumococcal antigens, and a respiratory viral panel. Covid-19 and serum HIV testing were negative.

## Outcome and follow up

He made a prompt recovery with a course of intravenous piperacillin with tazobactam and stepped down from intensive care on day 4 of his admission. His chest tube drain was removed on day 6, and he was discharged on day 7 of his hospital stay (day 11 from initial presentation) with a 4-week course of oral amoxicillin with clavulanic acid.

At a pleural outpatient clinic review 1 month after discharge, it was established that he was exposed to artificial stone and other rock dusts in his role as a Computerized Numerical Control (CNC) stone-cutting machine operator for a marble and granite supplier. He described working 8 hours a day per 5-day work week for the past 5 years adjacent to the CNC machine, mostly for artificial stone (engineered quartz) but also with natural quartz, granite, marble and porcelain. The machine used water suppression and the patient sometimes wore a filtering facepiece 3 (FP3) standard mask. There was an extractor fan but despite this, there would not infrequently be visible stone dust in the air, some of which arose as spill-over from an adjacent stone polishing room. He was an active smoker, starting at age 16 and having smoked 20–30 cigarettes per day prior to presentation (an estimated 22 cumulative pack years). Follow-up thoracic CT showed resolution of his right-sided empyema with residual pleuroparenchymal bands, and in addition, subtle diffuse pleural thickening and upper lobe-predominant centrilobular nodularity within the lungs consistent with simple silicosis that had not been appreciated previously. His lung function was within normal limits with spell out FVC 4.47 L (92% predicted), FEV_1_ 3.62 L (89%), ratio 81%; his TLCO, however, was reduced to 66.5% of predicted. A diagnosis of silicosis was made on the basis of the occupational exposure history and CT findings of nodular disease. Screening for TB (TB elispot test) and autoimmune disease (ANA, rheumatoid factor, C3 and C4 levels) was negative.

## Discussion

Both synthetic and natural stones contain varying concentrations of crystalline silica with relatively high levels of respirable dust generated through cutting operations such as those performed by the patient. Of the materials used, synthetic (engineered) stone can contain much higher levels than common natural decorative stones such as granite and marble [5]. Recent use of this popular material has been linked to outbreaks of silicosis, including many persons with advanced disease, reported in Israel, Australia, Spain and the USA [6].

Cumulative silica exposure is associated with macrophage dysfunction and increased risk of respiratory tract infection [7]. This phenomenon is recognized clinically in both mycobacterium tuberculosis and in atypical mycobacterial disease, and epidemiologically in bacterial pneumonia in persons exposed to metal fumes and dusts, including silica [3].

Empyema usually follows bacterial pneumonia, with streptococcus pneumonia being a common causal organism. Pneumococcal pneumonia carries a prominent occupational attributable risk among working-age adult males [8]. The incidence of empyema has been increasing in the UK, particularly in older people; the cause for this is unknown. Boys and girls are affected roughly equally up until age 15, after which the M:F ratio begins to increase reaching approximately 2.5:1 among those over age 60 [9]. One explanation for this sex difference could be differing occupational exposures among males.

In this case, the precipitating bacterial pneumonia is presumed, but could not be established by culture nor by DNA-based testing, most likely because of prior antibiotic use. A recent retrospective study found that culture-negative empyema occurred in about 20% of empyema cases with frank pus [10]. In a Swedish study of 745 patients with culture-positive invasive pneumococcal disease, pneumonia with empyema was twice as common (15% versus 7%) among previously healthy patients, even though the majority of all patients (70%) had comorbidities; smoking was common among both patients with (58%) and without (56%) comorbidities [11].

We could not identify any published cases of empyema being attributed to silica. We did find five cases of non-purulent culture-negative pleural effusion being attributed to silicosis [12–16]. Beyond clinical case reports, there also is an epidemiological association between silica exposure and pleural effusion without infection. A retrospective study of 110 patients with biopsy-proven silicosis found that 12 (11%) had pleural effusion on chest radiograph or CT and pleural thickening was found in 64 (58%) of patients at CT [17]. We identified only one report of occupational empyema, in an otherwise healthy, non-silica-exposed pickle seller whose aspirate grew *Leuconostoc mesenteroıdes*, a bacterium common in the pickling process [18].

It seems most likely that heavy silica exposure, together with heavy smoking, predisposed this otherwise healthy patient to pneumonia and, in turn, empyema. Further study should be made of pneumonia and empyema in silica-exposed workers to better understand the occupational burden of disease and to inform prevention efforts.
